# Intertwined Epidemics: National Demographic Trends in Hospitalizations for Heroin- and Opioid-Related Overdoses, 1993–2009

**DOI:** 10.1371/journal.pone.0054496

**Published:** 2013-02-06

**Authors:** George Jay Unick, Daniel Rosenblum, Sarah Mars, Daniel Ciccarone

**Affiliations:** 1 School of Social Work, University of Maryland, Baltimore, Maryland, United States of America; 2 Department of Economics, Dalhousie University, Halifax, Nova Scotia, Canada; 3 Department of Family and Community Medicine, University of California San Francisco, San Francisco, California, United States of America; The Scripps Research Institute, United States of America

## Abstract

The historical patterns of opiate use show that sources and methods of access greatly influence who is at risk. Today, there is evidence that an enormous increase in the availability of prescription opiates is fuelling a rise in addiction nationally, drawing in new initiates to these drugs and changing the geography of opiate overdoses. Recent efforts at supply-based reductions in prescription opiates may reduce harm, but addicted individuals may switch to other opiates such as heroin. In this analysis, we test the hypothesis that changes in the rates of Prescription Opiate Overdoses (POD) are correlated with changes in the rate of heroin overdoses (HOD). ICD9 codes from the Nationwide Inpatient Sample and population data from the Census were used to estimate overall and demographic specific rates of POD and HOD hospital admissions between 1993 and 2009. Regression models were used to test for linear trends and lagged negative binomial regression models were used to model the interrelationship between POD and HOD hospital admissions. Findings show that whites, women, and middle-aged individuals had the largest increase in POD and HOD rates over the study period and that HOD rates have increased in since 2007. The lagged models show that increases in a hospitals POD predict an increase in the subsequent years HOD admissions by a factor of 1.26 (p<0.001) and that each increase in HOD admissions increase the subsequent years POD by a factor of 1.57 (p<0.001). Our hypothesis of fungibility between prescription opiates and heroin was supported by these analyses. These findings suggest that focusing on supply-based interventions may simply lead to a shift in use to heroin rather minimizing the reduction in harm. The alternative approach of using drug abuse prevention resources on treatment and demand-side reduction is likely to be more productive at reducing opiate abuse related harm.

## Introduction

Opiates, the paradigmatic drugs of addiction, have caused medical concern for over a century and accidental self-poisoning for much longer [1]. For white upper and middle class users in the nineteenth century, preparations of opium and morphine were widely supplied by physicians who played an intimate part in the spread of addiction. The isolation of morphine in 1805 and the widespread uptake of the hypodermic syringe had expanded the possibilities for doctors to treat common symptoms, unintentionally turning many of their patients into addicts [2], [3]. As prohibition ruled out sanctioned use in the early twentieth century, nonmedical heroin use and overdose deaths shifted to marginalized groups [4]. These historical patterns show that sources and methods of access to opiates greatly influence who is at risk. Today there is evidence that an enormous increase in the availability of prescription opioids is fuelling a rise in addiction nationally, drawing in new initiates to these drugs and changing the geography of opiate-related overdoses [1], [5].

Emblematic of this new cycle of opiate misuse is the dramatic rise in unintentional drug poisonings which currently represent the second leading cause of injury related death in the United States and the leading cause of death for 35 to 54 year olds [6]. Prescription opioid-related overdoses represent the largest source of unintentional drug poisonings and their incidence has been increasing since the early 1990s [7]–[10]. This increase in prescription opioid-related overdoses has led to an increase in health care utilization that is troubling for both its high social and health care related costs [11]–[13].

Most of the current cases of opiate-related overdose can be traced to two fronts: that of illegal heroin consumption and the illicit use or misuse of prescription opioids, such as hydrocodone or oxycodone [8], [11]. Given the differences in the sources of opiates and the financial and social costs associated with overdose cases, it is important to understand the dynamics of the opiate-related overdose epidemic and the changing characteristics of opiate abusers.

The few recent studies that have investigated the dynamics of opiate-related overdose have focused solely on the emergence of the prescription opioid-related overdose epidemic. Evidence from the Drug Abuse Warning Network (DAWN) data shows that there has been a significant increase in the number of Emergency Department admissions associated with oxycodone and hydrocodone [7]. Particularly alarming is the rise in prescription opioid-related overdose deaths, especially in rural areas; between 1999 and 2004 prescription opioid-related overdose deaths increased 52% in large urban counties in contrast to 371% in non-urban counties [10]. The authors attribute this increase to the growing illicit use of prescription opioids that are widely available in rural areas. However, in selective areas heroin-related overdose constitutes a substantial proportion of total opiate-related overdose cases [14]–[16].

Further evidence of a changing dynamic of opiate abuse comes from CDC reports suggesting that prescription opioid-related overdoses rates are increasing the most in middle age individuals [17]. There are also hints that the racial and ethnic dynamic of the opiate use is changing. Prescription opioid abuse involves more White and less urban populations than previous opiate, i.e., heroin epidemics. Interestingly, evidence from the health disparities literature suggests that white patients have more access to prescription opioids than African American patients [18]. Similarly, trends in injecting drug using populations suggest shifts in usage patterns with young African Americans less likely to inject drugs while young whites represent a growing proportion of treatment seeking injectors [19].

There is contemporary evidence of an evolving flux between prescription opioid use and heroin use in urban areas. Ethnographic work from Montreal shows that individuals historically at risk for heroin injection have begun shifting to injecting prescription opioids [20]. Additional evidence from Baltimore and Washington State suggests high prevalence of prescription abuse in injection drug using populations, often preceding the initiation of heroin use [21], [22]. Interestingly, recent changes to the formulation of *OxyContin*, the brand name long-acting form of oxycodone, have been linked to a self-reported rise in heroin use [23]. What these studies suggest is that prescription opioid misuse and heroin use are *intertwined*.

While the increase in prescription opioid-related overdoses has been widely reported, the bulk of this documentation has occurred in government publications with few national longitudinal studies detailing the changing demographic characteristics of opiate-related overdose cases. Furthermore, no studies to our knowledge have investigated the relationship between prescription opioid-related overdoses and heroin-related overdoses using national data. The aim of this paper is to document changes in the demographics and examine the association between prescription opiate and heroin-related overdoses.

This study uses a consecutive series of nationally representative samples of hospital admissions from 1993 to 2009 to understand the changing demographics of prescription opioid-related overdoses (POD) and heroin-related overdoses (HOD) and to measure associations between changes in POD and HOD in hospitals. Hospital-based admission data have the benefit of focusing only on severe cases that have been vetted, generally in an emergency room, using more uniform standards and better ICD-9 coding of conditions than emergency room admissions. While more uniform, hospital admissions limit the sample to only to most severe cases. The large representative national sample allows for precise estimates of the extent of hospital admissions related to opiates in both urban and rural areas and among different demographic groups.

## Methods

Data come from the Nationwide Inpatient Sample (NIS), Healthcare Cost and Utilization Project (HCUP), Agency for Healthcare Research and Quality [24]. The NIS is an approximately 20-percent stratified national sample of United States Community Hospitals. The 1993 through 2009 NIS trends files were used to estimate the number of POD and HOD hospitalizations, consistent with HCUP’s recommendation that longitudinal use of the NIS start in 1993 because of changes in the sample [25]. Cases of POD and HOD were coded using ICD-9 codes included in the billing records reported by participating states to the NIS. An admission was considered a POD or HOD if one of the two following conditions was met: a primary diagnosis of 965.00, 965.01, 965.02 or 965.09, or an E code of E850.0, E850.1 or E850.2. PODs were restricted to primary diagnoses coded as 965.00, 965.02 or 965.09 or an E code of E850.1 or E850.2. HODs were restricted to primary diagnoses coded as 965.01 or an E code of E850.0.

SAS Software version 9.2 was used to code the data. For the analysis of trends in the demographics of POD and HOD, SAS Proc Surveyfreq was used to estimate the number of admissions adjusting the estimates for the sampling design using weights, strata and sampling units. SAS Proc Reg was used for the regression analysis of trends and SAS Proc Means was used if there was no statistically significant trend in the rates.

Rates of POD and HOD hospitalizations per 100,000 US population were estimated for years 1993 through 2009. Rates based on cross tabulations of POD and HOD with race, gender, and age categories in five-year increments were calculated per 100,000 members of the respective demographic group per year between 1993 and 2009. We used a weighted moving mean smoothing with the prior year and post year weighted as a one and the current year weighted twice for all the HOD data displayed in the figures. The smoothing was performed to reduce the noise in the HOD data that made it difficult to see underlying trends. We used raw HOD data for all the other analyses. These rates were used to test for linear and quadratic trends over time. A nested series of models were estimated for each series of rates. All year means were estimated, followed by a sequence of regression models with a linear, quadratic (time^2^) and cubic (time^3^) coefficients estimated. A single linear time coefficient represents a straight line. Quadratic and cubic coefficients allow the slope of the line to change over time. Quadratic coefficients allow for a single rate of change in the slope of the line such as an increase in the rate of overdoses or a decrease in the rate of overdoses. A cubic coefficient allows for wave-like changes in the slope of the line, suggesting alternating periods of increasing, flat and decreasing rates. Means are only shown when the time trends were nonsignificant. When two or more of the linear, quadratic and cubic models were statistically significant the models with the highest R^2^ are showed. Statistical significance for the quadratic or cubic models represents the combined statistical significance of all the time variables. Means and standard deviations are reported in cases where there are no statistically significant trends.

For the analysis of the interrelationship between POD and HOD, Stata 12 was used to estimate negative binomial lag models. Variables were constructed for prior year POD and HOD. Two negative binomial lag models were estimated, one associating current year raw hospital counts of HOD and another associating counts of POD, as functions of the previous years (one year lag) count of HOD and POD, along with time and the urbanicity of the hospital as covariates. Urbanicity of hospitals is constructed by HCUP from the US Department of Agriculture urban rural coding [26]. Cluster robust standard errors were used to account for the repeated measures nature of the data and for the minor violations of distributional assumptions about the amount of hospitals with zero ODs in the data.

## Results

**Figure 1:**
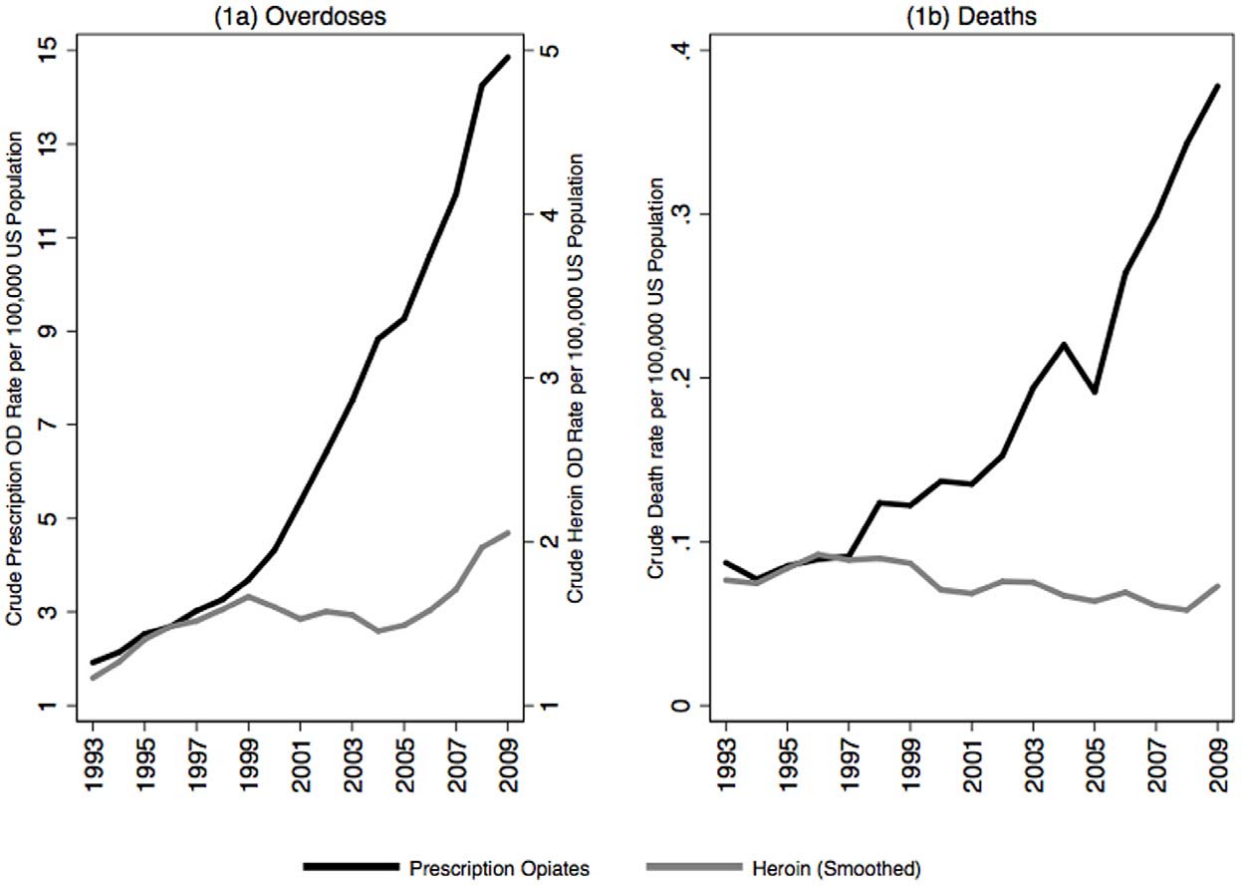
Rates of overdose and death.

Figure 1 displays changes in the rate of hospitalization for POD and HOD and deaths per 100,000 US population per year. Figure 1a shows that the rate of increase between POD and HOD is roughly equivalent until 1997, but subsequently diverges, with the rate of POD geometrically increasing. HOD admissions show a wave-like pattern with an increase between 1993 and 1999 and a slight decrease between 2000 and 2005, followed by an increase starting in 2006. The acceleration in the rate of POD is seen in the ratio of POD to HOD: the ratio is about 2 in 1997, rising to 7.6 in 2009. Table 1 displays the linear, quadratic or cubic models for each of the trends. Consistent with Figure 1, there is evidence for a significant cubic or wave-like pattern in HOD between 1993 and 2009 driven in large part by the substantial increase in HODs since 2006. The POD model has a large and statistically significant quadratic coefficient for POD, indicating an accelerating rate of overdose admissions shown in Figure 1.

**Table 1:** Trends analysis.

Variable	Lowest Rate	Highest Rate	Intercept/mean (SD)	Linear	Quadratic	Cubic	Adj R^2^
Heroin OD	1.13 (1993)	2.26 (2008)	1.08	0.23	−0.03	.001**	0.54
Prescription OD	1.92 (1993)	14.85 (2009)	2.04	0.022	0.05***	–	0.99
Heroin Death	0.06 (1994)	0.09 (2009)	0.08 (0.02)	–	–	–	–
Prescription Death	0.08 (1994)	0.38 (2009)	0.09	−0.004	.001***	–	0.97
White HOD	0.68 (1993)	1.91 (2009)	0.58	0.28	−0.04	0.002***	0.73
Black HOD	1.12 (2005)	3.62 (1995)	2.28	0.52	−0.10	0.004***	0.78
Hispanic HOD	0.79 (2008)	2.15 (1995)	1.54	0.31	−0.05	0.002***	0.75
White POD	1.95 (1993)	16.51 (2009)	2.52	−0.19	0.06***	–	0.99
Black POD	1.80 (1993)	7.76 (2009)	1.94	0.28	−0.03	.002***	0.96
Hispanic POD	1.06 (1993)	4.42 (2009)	1.32	0.001	0.01***	–	0.95
Male HOD	2.00 (2005)	3.33 (2008)	2.04	0.29	−0.04	0.002***	0.87
Female HOD	0.64 (1993)	1.26 (2008)	0.62	0.13	−0.08	0.001***	0.78
Male POD	2.08 (1993)	13.73 (2009)	2.27	0.04	.04***	–	0.99
Female POD	2.44 (1993)	15.93 (2009)	2.39	0.09	.05***	–	0.99
20 to 24 HOD	1.38 (1994)	5.11 (2009)	1.16	0.83	−0.10	0.004***	0.76
25 to 29 HOD	2.48 (2001)	4.75 (2009)	2.68	−0.05	0.009***	–	0.70
30 to 34 HOD	2.43 (2005)	4.64 (2000)	3.33 (0.55)	–	–	–	–
35 to 39 HOD	2.05 (2009)	4.04 (1997)	4.03	−0.09	−0.001***	–	0.63
40 to 44 HOD	2.07 (2007)	4.86 (2008)	3.11 (0.70)	–	–	–	–
45 to 49 HOD	1.07 (2007)	4.22 (2008)	2.67 (0.64)	–	–	–	–
50 to 54 HOD	0.60 (1993)	3.59 (2008)	0.70	0.17	−0.003***	–	0.65
55 to 59 HOD	0.35 (1993)	1.94 (2008)	0.36	0.15	−0.02	0.001***	0.65
60 to 64 HOD	0.18 (2003)	0.87 (2009)	0.46 (0.24)				
20 to 24 POD	2.43 (1994)	12.05 (2009)	2.36	−0.05	0.04***	–	0.98
25 to 29 POD	2.72 (1993)	13.47 (2008)	2.86	−0.30	0.05***	–	0.97
30 to 34 POD	3.06 (1993)	15.63 (2009)	3.37	0.14	0.04***	–	0.97
35 to 39 POD	4.06 (1994)	15.14 (2009)	4.02	0.28	0.03***	–	0.98
40 to 44 POD	2.89 (1993)	17.93 (2008)	3.12	0.65	0.02***	–	0.98
45 to 49 POD	2.44 (1993)	23.65 (2009)	1.97	0.69	0.04***	–	0.99
50 to 54 POD	1.82 (1993)	30.60 (2009)	2.12	−0.06	0.12***	–	0.99
55 to 59 POD	1.57 (1993)	26.51 (2009)	2.63	−0.30	0.11***	–	0.99
60 to 64 POD	2.15 (1994)	23.74 (2009)	2.70	−0.31	0.10***	–	0.99

* p<0.05

** p<0.01

*** p<0.001.

Figure 1b shows corresponding trends in POD and HOD deaths from 1993–96 but with a sharp divergence starting in 1997. HOD deaths peaked in 1999 with a rate of 0.11 per 100,000, reaching a low point in 2008 at 0.04 per 100,000. In contrast, POD deaths had their low point in 1994 at 0.08 per 100,000, increasing exponentially to 0.38 per 100,000 in 2009.

**Figure 2:**
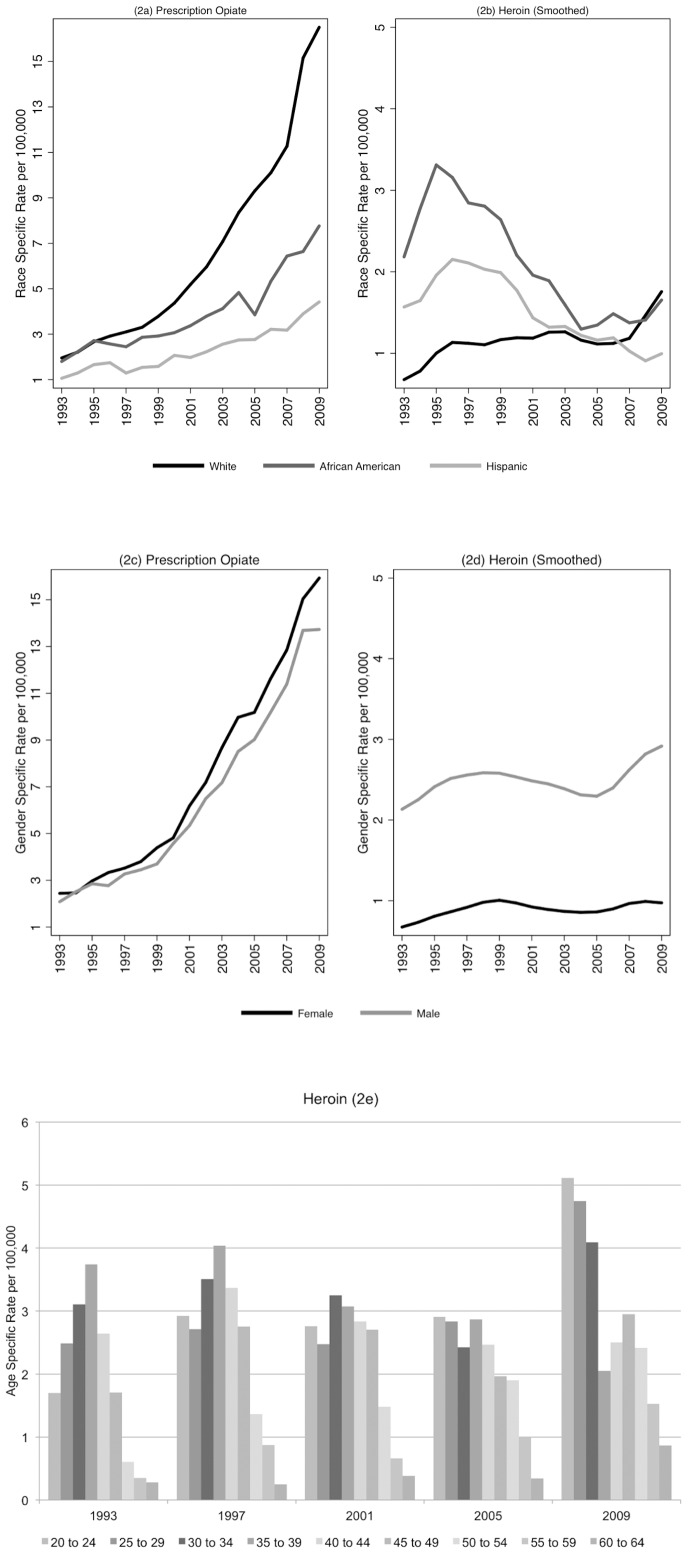
Demographic trends.

Figure 2 shows that changes in POD and HODs are not equally distributed in the population. Figure 2a shows changes in the rates of PODs for the 3 most common racial and ethnic categories. Between 1993 and 2009 POD increased for all race/ethnic groups but at different rates of increase. The rate of POD admissions for whites increased 7.5 times; more than double that for African Americans (3.3) and Hispanics (3.2).

Figure 2b shows that HOD admissions among African Americans decreased from a peak race-adjusted rate of 3.62 per 100,000 in 1995 to a low rate of 1.12 in 2005. (Table 1) The rate of HOD admissions among African Americans trends significantly downward during most of the study period, with an uptick in 2009. Similarly, the race-adjusted rate of admissions for Hispanics decreased during the study period. The peak rate of HOD for blacks and Hispanics occurred in 1995 with the lowest rates in 2005 and 2008 respectively. HOD admissions for whites showed a statistically significant increase between 1993 and 2009, with the cubic model suggesting an increase between 1993 and the early 2000, followed by a brief flattening and then a rapid uptick in the late 2000s. HOD among whites hit a nadir in 1993 with a race-adjusted rate of 0.68 per 100,000 whites, peaking in 2009 at a race-adjusted rate of 1.91. In 2008, HOD hospitalization rates for whites exceed that of African Americans for the first time since 1993.

Figure 2c and 2d show overdose trends by gender. Both female and male trends in PODs reveal significant quadratic increases. The linear trend in the gender specific HOD graphs is similar to the overall trend in HODs. In contrast to HODs, women have higher rates of PODs compared to men for all study years.

Table 1 provides evidence for a consistent increase in the age-adjusted rates of POD admissions; with the lowest rates in 1993/4 and the highest in 2008. In all cases presented here positive quadratic coefficients suggest rapid increases in rates over time, even if the linear coefficients are negative. All age groups show significant positive quadratic increases in the rates of PODs between 1993 and 2008, indicating that increases in admissions for all age groups accelerated during the 2000s. In contrast HOD admissions have more heterogeneity across age categories. Rates of HOD admissions decreased or were flat between 1993 and 2009 for all age groups between 30 and 49, while, age groups between 20 to 29 and 50 to 59 had statistically significant increases in rates of HOD hospitalizations, suggesting the simultaneous aging of the heroin using population and perhaps – the emergence of a new cohort of heroin users. Figure 2e shows a series of snapshots of the age distribution of individuals hospitalized for HOD at four year intervals. What is striking about figure 2e is the large increase in 2009 for HODs in 20 to 34 year old age groups.

Table 2 shows the results of the lagged negative binomial (NB) regressions, modeling the hospital counts of POD and HOD. The first model measures the correlates of POD hospital counts using the one year lagged HOD counts, the urbanicity of the hospital, and an interaction between hospital urbanicity and the lagged HOD while controlling for a one year lagged count of PODs and time. The incident rate ratio (IRR) shown in table 2 suggests that there is an association between HOD and POD. Specifically, holding all other variables constant, each HOD increases the subsequent year’s rate of PODs by a factor of 1.57 (p<0.001). Stated another way, a one standard deviation increase in the prior year’s HODs, approximately 4 overdoses, results in an expected mean increase of 205.6% in the subsequent year’s PODs after accounting for the prior rate of PODs at that hospital. Urban hospitals have a 51.9% (p<0.001) higher expected mean rate of PODs, holding other variables constant. The relationship between the lagged HOD and current year PODs is 40% (p<0.001) weaker in urban hospitals compared with rural hospitals.

**Table 2:** Negative Binomial Lag Models for PODs and HODs.

POD Model	IRR	95% CI
Time	1.05***	(1.05, 1.06)
POD Lag	1.16***	(1.14, 1.18)
HOD Lag	1.57***	(1.40, 1.76)
Urban Hospital	2.32***	(2.10, 2.56)
Urban Hospital x HOD Lag	0.66***	(0.59, 0.74)
Constant	0.47***	(0.43, 0.52)
HOD Model	IRR	95% CI
Time	0.96***	(0.94, 0.97)
POD Lag	1.26***	(1.20, 1.33)
HOD Lag	1.38***	(1.33, 1.44)
Urban Hospital	8.42***	(6.42, 11.04)
Urban Hospital x POD Lag	0.84***	(0.80, 0.89)
Constant	0.07***	(0.05, 0.09)

*** p<0.001.

The HOD model uses a one year lagged count of hospital POD, urbanicity of the hospital and interaction between urbanicity and POD, while controlling for the a one year lagged count of HOD and secular trends using a time coefficient. This model suggests that there is a strong relationship between PODs and HODs. Holding all other variables constant, each additional OD in the previous year’s hospital count of POD increases the hospital’s rate of the subsequent year HOD by a factor of 1.26 (p<0.001). A standard deviation increase in the prior year’s PODs, roughly equivalent to 7 overdoses, increases the mean expected count of HODs for a hospital by 193%. As expected urban hospitals have higher rates of HOD compared with rural hospitals by a factor of 8.42 (p<0.001) holding all other variables constant. As with the POD model, the influence of the one-year lagged PODs on the current year’s count of HODs is 15% (p<0.001) greater in rural hospitals compared with urban hospitals.

## Discussion

The findings presented here are consistent with the existing literature describing the exponential growth of POD between 1993 and 2009. However, we also document the recent increase in HOD and provide initial evidence suggesting a link between the growth of PODs and the recent surge of HODs. While preliminary and not evidence of a causal relationship, the findings presented here provide empirical support for a growing concern about the commingling of prescription opioid and heroin abuse.

Consistent with the literature, we find changes in the populations most at risk for this recent opiate-related overdose epidemic. The most dramatic increases in POD are seen among whites, women (with rates exceeding that of men) and the middle-aged. Consistent with the hypothesis that prescription opioid use is creating new users of heroin, the race-adjusted rate of HOD has doubled among whites, exceeding in 2008 that of African-Americans for the first time, while also significantly increasing among 20–24 year-olds – an age group at high risk for escalating substance abuse. The rate of HOD has declined among African-Americans, perhaps reflecting generational resilience following the devastating effect of the 1960–70′s heroin epidemic on African-American communities.

The acceleration in HOD admission rates after 2006 raises fresh concerns. Previous literature declared that HOD was mostly flat during the unfolding of the prescription opioid epidemic [27]. Our data reveal that from 1993 to 2006 the rate of HOD rose 69% with much of the increase occurring at the recent end of the data: from 2005 to 2009 the HOD rate increased 44%, or 11% per annum. The lagged regressions, modeling the subsequent years’ POD/HOD rates, demonstrate strong relationships between these adverse consequences. While it needs to be emphasized that these are not causal models, this finding is consistent with population level dynamics in which the same population of drug users is simultaneously using diverse opiates or migrating from one to another due to structural, market, or social forces. Indeed, the transition to heroin use is reported in several local press articles [28], [29].

While consistent with the above-presented explanation other hypothesis could explain these findings. For example, heroin use may follow a cyclical pattern with generational resilience and generational forgetting (i.e. the consequences) alternating. Various socio-economic causal mechanisms may also simultaneously be responsible for both forms of opiate epidemics with little need of intertwining between them. Furthermore, hospital data undercount overdoses that result in deaths or are otherwise treated in the community. These nonhospital deaths may have a different racial or ethnic composition due to differences in community risk and other behavioral factors [30]–[32]. Likewise, a lack of access to hospital or medical care may also have biased the sample, resulting in a under count of overdoses among racial or ethnic minority populations.

However, this recent experience is consistent with the historical evidence suggesting that changes in the form of opiates, their availability and the stigma attached to them coincides with changes in the population at risk for abuse and overdose. The recent rise in POD corresponds with the rise in prescription opioid availability. This in turn has been driven by a number of factors: increasing supply, pain management advocacy, pharmaceutical marketing directed to prescribers, and drug diversion. Drug diversion is a particular problem exacerbated by doctor shopping, internet sales, theft, and improper prescribing [33]. Retail sales have grown 533% from 1997–2005 with hydrocodone the leading prescribed medication and oxycodone the top retailed prescription opioid by weight [34]. *OxyContin* was approved in 1995 and sales rose rapidly, increasing 69-fold from $44.8 million in 1996 to $3.1 billion in 2010 [35], [36]. The Drug Enforcement Agency expressed concern about the aggressive marketing of *OxyContin* to physicians and responses to the prescription opioid misuse problem have been growing in number and strength since 2003 [35]. These include regulatory responses (drug monitoring programs and state disciplinary actions), increasing physician awareness of appropriate treatment practices and modifying drugs to reduce their potential for abuse [37]. One noteworthy response followed a 2007 court case brought against the manufacturer of brand name long-acting oxycodone (*OxyContin*) to which it plead guilty to falsely misrepresenting the addictive qualities of the drug compared with other pain medications [38]. In the face of widespread criticism, the tablets were reformulated into a tamper proof gel tablet and approved by the FDA in 2009 [39].

Reversal of the “pendulum swing” from under-treatment of pain to over-treatment may have intended (e.g., improved treatment of chronic pain with non-opioid modalities) and unintended consequences (e.g., former patients seeking illegitimate opiates, e.g., diverted prescription opioids or heroin) [40]. Heroin use has some fluidity among certain populations and injectors may switch between opiates [41]. There is a stigma threshold for heroin injection that initial prescription opioid misuse may facilitate. Once dependent, some prescription opioid misusers learn to crush, insufflate or inject their prescription opioid of choice prior to seeking heroin. Polydrug use is common among rural substance users with heroin use independently associated with prescription opioid misuse [42].

Preliminary observations from a companion qualitative study in inner-city Philadelphia reveal some of the market incentives for users transitioning from prescription opioids to heroin. Heroin is inexpensive and pure by historical standards and long-acting oxycodone is typically more expensive than heroin [43]. Consider this rough calculation: in the early 2000’s street diverted *OxyContin* OC (crushable form) cost ∼$0.50 per mg (unpublished data) and heroin has had a mean street-price of $0.56/mg-pure [43]; seemingly similar until one considers that the parenteral equivalent dose of heroin is 1/2 to 1/3 that of oxycodone. Furthermore, *OxyContin* is now less desired as a street drug in its new tamper-resistant formulation [44].

There are several limitations to this study. The reliance on hospital coding for accurate diagnosis of the cause of opiate-related overdose induces some unaccountable variability. For example, hospitals may use a single code for all opiate-related overdoses, combining heroin and prescription opiates, making it difficult to distinguish specific drugs responsible for an overdose admission. Using hospital admissions rather than emergency room admissions also may affect the population by only selecting cases with more serious medical conditions. This sampling frame may result in a population that is older and with a higher prevalence of comorbid medical conditions than the opiate using population in general. Many opiate-related overdoses can be treated effectively in the ED regardless of the amount of opiate consumed and different hospitals may be more effective in treating ODs in the ED than other hospitals. We also cannot distinguish between overdoses resulting from illicit use, misuse or accidental use. Finally, using hospital admissions rather than individuals as the units of analysis we cannot control for individual level covariates or examine repeat visits in the lag models. While the degree to which the biases are unknown suggests caution in interpreting the results, the large representative sample of hospitals and random nature of many of these potential sources of errors suggests that the effect of these limitations is likely modest.

Given the dramatic increase in the rate of POD and the strong potential for current structural reforms aimed at reducing prescription opioid misuse to inadvertently shift a proportion of prescription opioid users to heroin, a robust public health response is necessary. Active surveillance looking for a rise in heroin use/users is a good starting point. Public health measures that can address and reduce overdose deaths should include primary overdose prevention e.g. treatment of substance use, including opiate substitution therapies i.e. methadone and buprenophine. Buprenorphine, a partial opiate agonist successful in treating heroin dependency [45], should be expanded to the wide variety of opiate dependent users.

Secondary prevention efforts should include national opiate overdose awareness campaigns; these could be coupled to national policies addressing prescription drug abuse [46]. Tertiary overdose prevention efforts should include targeted campaigns to recognize and reverse overdose including harm reduction-based peer interventions such as rescue breathing, naloxone administration, and safe injection facilities. Evidence for the effectiveness of peer-based naloxone interventions is gathering on the local, county and statewide levels[47]–[55]. Indeed the stable HOD death rate compared with the rising hospitalization rate noted in this study may be suggestive of evidence for the national uptake and success of these interventions, albeit with uneven adoption and financial concerns [56].
